# Neural Correlates of Causal Inferences in Discourse Understanding and Logical Problem-Solving: A Meta-Analysis Study

**DOI:** 10.3389/fnhum.2021.666179

**Published:** 2021-06-23

**Authors:** Wangshu Feng, Weijuan Wang, Jia Liu, Zhen Wang, Lingyun Tian, Lin Fan

**Affiliations:** ^1^Research Institute of Foreign Languages, Beijing Foreign Studies University, Beijing, China; ^2^National Research Centre for Foreign Language Education, Beijing Foreign Studies University, Beijing, China; ^3^Artificial Intelligence and Human Languages Lab, Beijing Foreign Studies University, Beijing, China

**Keywords:** causal inferences, meta-analysis, neuroimaging, discourse understanding, logical problem-solving, frontotemporal network

## Abstract

In discourse comprehension, we need to draw inferences to make sense of discourse. Previous neuroimaging studies have investigated the neural correlates of causal inferences in discourse understanding. However, these findings have been divergent, and how these types of inferences are related to causal inferences in logical problem-solving remains unclear. Using the activation likelihood estimation (ALE) approach, the current meta-analysis analyzed 19 experiments on causal inferences in discourse understanding and 20 experiments on those in logical problem-solving to identify the neural correlates of these two cognitive processes and their shared and distinct neural correlates. We found that causal inferences in discourse comprehension recruited a left-lateralized frontotemporal brain system, including the left inferior frontal gyrus, the left middle temporal gyrus (MTG), and the bilateral medial prefrontal cortex (MPFC), while causal inferences in logical problem-solving engaged a nonoverlapping brain system in the frontal and parietal cortex, including the left inferior frontal gyrus, the bilateral middle frontal gyri, the dorsal MPFC, and the left inferior parietal lobule (IPL). Furthermore, the pattern similarity analyses showed that causal inferences in discourse understanding were primarily related to the terms about language processing and theory-of-mind processing. Both types of inferences were found to be related to the terms about memory and executive function. These findings suggest that causal inferences in discourse understanding recruit distinct neural bases from those in logical problem-solving and rely more on semantic knowledge and social interaction experiences.

## Introduction

Discourse is a unit of language larger than a single sentence, which is mainly used for communication in speech or writing. In discourse understanding, people often need to draw inferences to build semantic coherence and to make sense of discourse (Van Dijk and Kintsch, 1983; Kintsch, 1988). Although the internal relations between the sentences or clauses within a discourse could be different, discourse comprehension has been found to mainly rely on inferring causality (Schank, 1975; Warren et al., 1979; Graesser and Clark, 1985; Keenan et al., 1990; Singer, 1994). Such causally relevant inferences, so-called causal inferences, allow the hearers and readers to supply the causes or effects that are not overt in the discourse and to establish the links of events or information between one utterance and another (van den Broek, 1990, 1994). Thus, making causal inferences is essential for daily communication in both spoken and written language.

In the past few decades, previous studies have investigated the cognitive and neural mechanisms of causal inferences across sentences during discourse comprehension using behavioral methods and neuroscience tools such as functional MRI (fMRI) and PET. Behavioral methods were adopted to explore various types of inference processes during real-time discourse comprehension, including predictive inferences (Calvo and Castillo, 2001; Calvo et al., 2001), bridging inferences (Sansosti et al., 2013), and elaborative inferences (Calvo et al., 2003). The results revealed that individuals could generate inferences online by activating and integrating textual information and background knowledge. In addition, substantial research examined the individual factors affecting inferential processing during text reading, such as comprehension skills (e.g., Hawelka et al., 2015), vocabulary knowledge (Calvo et al., 2003; Calvo, 2005), and working memory capacity (Calvo, 2005; Yeari, 2017).

Neuroimaging studies employing fMRI and PET techniques investigated the neural activities of causal inferences during discourse comprehension. One line of studies has explored inferential processing by comparing the stories with implicit causality to the stories with explicit causality (e.g., Kuperberg et al., 2006; Virtue et al., 2006, 2008; Siebörger et al., 2007; Mason and Just, 2011; Kim et al., 2012; Prado et al., 2015). For example, Kuperberg et al. (2006) conducted an fMRI experiment to measure the localization of neural activity-mediating causal inferences when a sentence was highly causally related, intermediately related, or unrelated to its preceding contexts. When compared to a highly related condition, the sentences intermediately related to the preceding contexts elicited increasing neural activities within the bilateral inferior frontal gyri (IFG), bilateral inferior parietal lobules (IPLs), left middle frontal gyrus (MFG), left middle temporal gyrus (MTG), and medial prefrontal cortex (MPFC). These results suggested that causal inferential processing may engage a semantic activation, retrieval, selection, and integration from the long-term memory during discourse comprehension. Virtue et al. (2006, 2008) aurally presented explicitly or implicitly stated inference scenarios to the participants and compared the differences of neural activity in the earlier (i.e., the verb that implied the inference) and later (the coherence break) time points. They confirmed the critical roles of the bilateral temporal cortex and found that the right superior temporal gyrus (STG) is particularly involved in early inferential processing while the left STG is particularly recruited in later inferential processing. Mason and Just (2011) distinguished different inference contents and revealed that a set of brain regions, including the MPFC, bilateral IFG, left posterior STG, and bilateral anterior temporal lobes, form a general inference network, while the right temporoparietal junction, as a theory-of-mind brain region, is involved in the inferences concerning others' intentions but not in physical inferences. Another line of studies on causal inferences has investigated elaborative or bridging inferential processing by directly comparing coherent discourse with incoherent discourse (e.g., Robertson et al., 2000; Ferstl and von Cramon, 2001, 2002; Kuperberg et al., 2006; Siebörger et al., 2007; Yarkoni et al., 2008; Prat et al., 2011). These studies also got results that were inconsistent and hard to collate. Concerning unrelated sentences, a coherent discourse may elicit activations in MPFC and the posterior cingulate cortex (Ferstl and von Cramon, 2001, 2002), activations in the frontoparietal areas related to executive functions (Siebörger et al., 2007), or activations in frontotemporal regions for situation model maintenance (Yarkoni et al., 2008). Different from the above two lines of studies, Chow et al. (2008) looked into the neural correlates of causal inferences by manipulating the reading goal (i.e., reading-with-prediction condition or reading-without-prediction condition). Comparing the reading-with-prediction condition to the reading-without-prediction condition, they found that explicitly predictive inferential processing elicited increased hemodynamic activity in the left anterior PFC and left anterior ventral IFG, reflecting the phase of coherence evaluation and the process of drawing strategic inferences. To sum up, previous studies found that the brain regions in both left and right hemispheres may be involved in the inference generation during discourse comprehension, especially the regions in the frontal and temporal lobes, but results for which region is involved in the process have been divergent.

The causally relevant inferences during discourse comprehension were often viewed as a problem-solving process (van den Broek, 1990) and were conceived by using an analogy to logical thinking (Suvorova and Polyakova, 2018). Meanwhile, considering the interplay between thought and language, an important view is that thinking is deeply rooted in the language (Polk and Newell, 1995). Previous studies have demonstrated that logical inferences relied heavily on language processing (Goel et al., 1998; Goel and Dolan, 2004). Thus, being one type of inferences outside the domain of discourse comprehension, causal inferences in logical problem-solving (hereafter “logical inferences”) seem to share some component processes with causal inferences in language understanding (hereafter “discourse inferences”). In this regard, we wondered whether the two types of inferences share some or even all cognitive processes.

Logical inferences are considered as a cognitive activity of evaluating arguments (Goel et al., 2004). On the basis of human rationality, the neural correlates of logical inferences have been investigated by using many neuroimaging studies. In these studies, participants were generally required to read sets of arguments rather than coherent texts and to evaluate whether the arguments are valid. Prado et al. (2011) performed a quantitative meta-analysis of 28 fMRI and PET studies on deductive reasoning. The results revealed that the studies consistently reported activations in the left frontoparietal network and in the left basal ganglia. Most recently, the meta-analysis of the conditional and syllogistic inferences including 32 original neuroimaging experiments, also showed that such causal inferences are associated with a left-lateralized widespread pattern of activations, which included the left (and right) IFG, left (and right) MFG, left IPL, left superior frontal gyrus, and MPFC (Wertheim and Ragni, 2020). Although the evidence from both abovementioned groups of studies has been accumulated, few studies have directly compared the neural correlates of discourse inferences and logical inferences. Hence, it is still an open question whether the neural bases of these two cognitive processes are similar or distinct.

In the current study, we aimed to investigate the shared and distinct neural bases of discourse inferences and logical inferences. We used meta-analytic methods to investigate the neural correlates of discourse inferences and logical inferences because meta-analyses could increase the statistical power and help to detect robust effects across the experiments. To address the aim, we first computed the map of each cognitive process using the activation likelihood estimation (ALE) method. In order to make data more comparable across those two lines of studies, we excluded the studies that used non-verbal stimuli as materials for logical inferences. Then, we compared them to investigate their conjunction and their respective specific activations. In addition, we conducted neural pattern similarity analyses to further identify the neurocognitive subprocesses underlying discourse inferences and logical inferences using a large-scale meta-analytic database. Based on the previous studies, we hypothesized that the discourse inferences may recruit the left frontotemporal network (e.g., Ferstl and von Cramon, 2001, 2002; Siebörger et al., 2007) and might even activate the regions in the right frontotemporal network (e.g., Kuperberg et al., 2006; Virtue et al., 2006, 2008; Mason and Just, 2011) while the logical inferences may engage the activations of a left-lateralized frontoparietal network (Prado et al., 2011; Wertheim and Ragni, 2020). Additionally, these two types of inferential processing may share the neural activity in IFG and MPFC.

## Materials and Methods

### Study Selection

**Figure 1:**
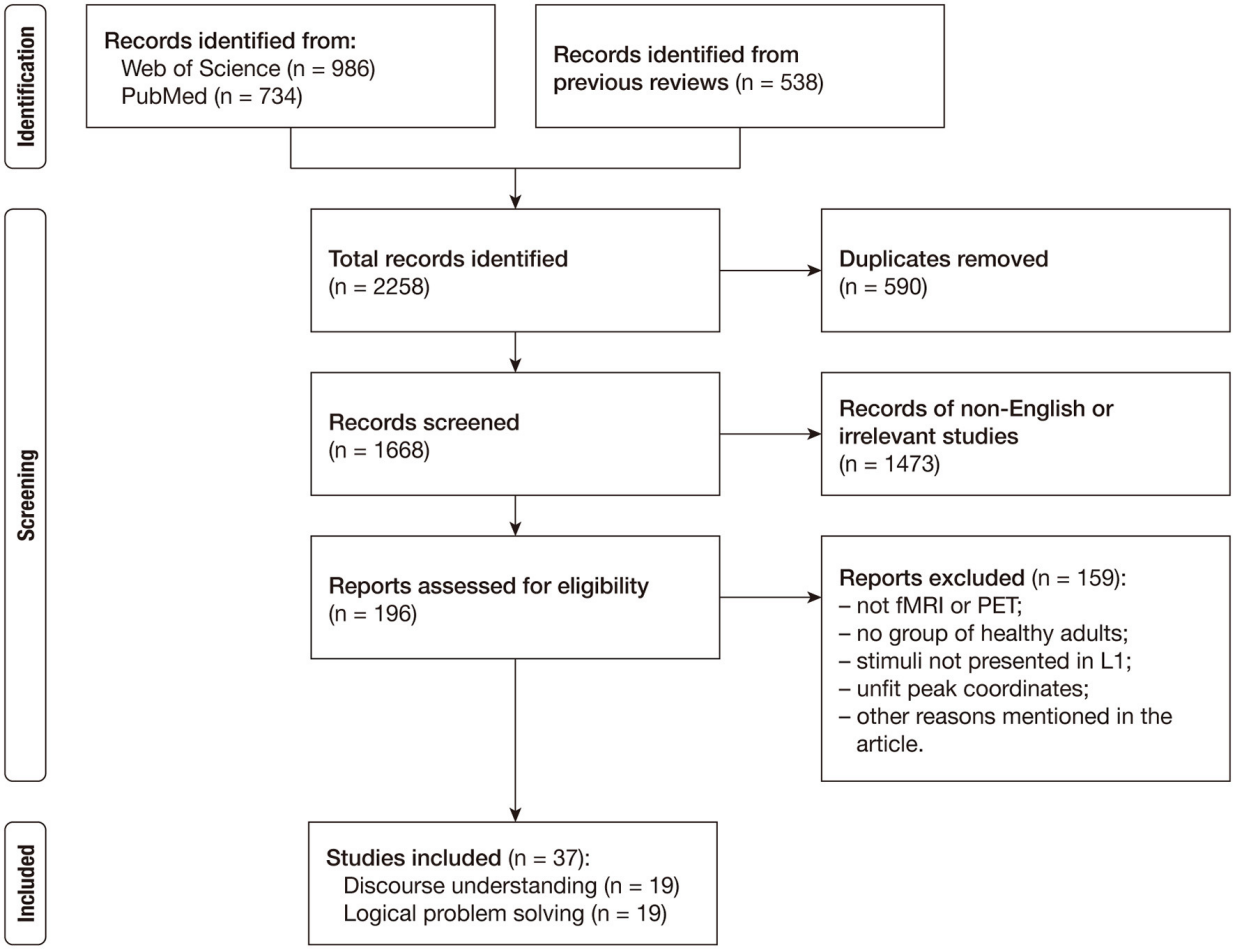
Flowchart of study selection.

The study selection process is presented in a flow diagram in Figure 1 and the definitions of key terms are provided in Table 1. We conducted an online literature search in the Web of Science and PubMed databases to acquire the studies published between January 1980 and March 2020 (last search on March 27, 2020). The function of the advanced search was used to detect the target literature. The primary literature was collected by using the following strategies: Topic = [(“causal^*^” OR “predict^*^” OR “bridg^*^”) AND (“inferen^*^” OR “reasoning”) AND (“fMRI” OR “neuroimaging” OR “functional magnetic imaging” OR “functional MRI” OR “functional MRI” OR “functional imaging”)]. This search generated 986 articles in the Web of Science database and 734 articles in the PubMed database. In order to avoid omission, we additionally considered the studies in the relevant meta-analyses (Prado et al., 2011; Yang et al., 2019; Wertheim and Ragni, 2020) and reviews (e.g., Barbey and Patterson, 2011; Virtue and Sundermeier, 2016; Brascamp et al., 2018; Wang et al., 2018), and the studies that cited those meta-analyses and reviews.

**Table 1:** Definitions and examples of key terms.

**Term**	**Definition**	**Example**
Causal inference	The process of determining causes and effects.	*Mark jabbed the balloon with the pin* implied the consequence that the balloon broke.
Logical reasoning	The process of drawing conclusions from premises or information.	*Julie had five apples and she gave one to Paul* implied that Julie ended up with four apples.
Inductive reasoning	A type of reasoning that synthesizes detailed facts or observations to reach general conclusions.	*The left-handed people I know use left-handed brush; therefore, all left-handed people use left-handed brush*.
Conditional reasoning	A type of reasoning that is based on the construct “if A, then B.”	*If today is Sunday, then I will not go to school. Today is Sunday, so I will not go to school*.
Syllogistic reasoning	A type of reasoning that draws a conclusion from two or more premises that are assumed to be true.	*All cats eat meat. Some animals are cats. Therefore, some animals eat meat*.
Bridging inference	The process of establishing connections between the current event and a prior text (or background knowledge).	*The patient's eyesight was restored painlessly, and the ophthalmologist liked the new method* implied that the ophthalmologist treated the eyes.
Predictive inference	The process of generating explanations about what will happen next in the discourse.	*Mark fell from the 14^*th*^ floor* implied the consequence that he was dead.
Elaborative inference	The process of extending or refining the explicit content in the discourse.	*Tomorrow is Mike's birthday. His girlfriend Jane went to a shopping mall* implied Jane's motive that she was going to buy him a present.

After deduplication, we removed the articles that were not written in English and the articles that were obviously irrelevant according to their title and abstract. Then, three of the present authors (JL, WW, and ZW) gathered relevant information from the remaining 196 studies, including the number of participants, research techniques, the type of tasks, the type of stimuli, the type of contrasts, and the standard space in which coordinates were reported. They checked each other's work, and they discussed and resolved a few disagreements. The studies included in the current meta-analysis must meet the following requirements: (1) the technology used was task-induced fMRI or PET; (2) the experiment recruited at least one group of healthy adult participants; (3) the stimulus materials were in the participants' first languages; and (4) peak coordinates were presented in the standard Talairach space or the standard Montreal Neurological Institute (MNI) space. We further excluded studies for their lack of univariate analysis at the whole-brain level (i.e., Mason and Just, 2004), their low-level baseline conditions (i.e., Knauff et al., 2003; Brunetti et al., 2014; Porcaro et al., 2014; Smith et al., 2014, 2015), or their usage of pseudoword or nonword (i.e., Prado and Noveck, 2007; Reverberi et al., 2010, 2012; Hearne et al., 2015). Moreover, studies on nonliteral language comprehension, such as conversational implicature or indirect speech, were not included in this meta-analysis (e.g., van Ackeren et al., 2012; Jang et al., 2013; Feng et al., 2017) because the processing involved in these complex pragmatic phenomena goes beyond inferring the causal structure of the literal meaning (see Bohrn et al., 2012; Rapp et al., 2012 for reviews). In addition, we focused on the comparison between an inference and a non-/weak-inference across the sentences; therefore, the studies that did not adopt sentence-level stimuli (i.e., Satpute et al., 2005; Wende et al., 2012) or did not include an appropriate contrast (i.e., Parsons and Osherson, 2001; Ferstl et al., 2005; Reverberi et al., 2007; Egidi and Caramazza, 2014, 2016) were excluded. Finally, the selected studies were classified as discourse inferences and logical inferences according to their verbal materials. Specifically, a study will fall into the logical inference group if the status of its stimuli depends on explicit logical connectors studied in elementary logic (e.g., *if…then, or, not*) while a study will fall into the discourse inference group if the inferential processing does not necessarily rely on the sentential connectives but mainly requires to build a semantic representation.

Overall, the current meta-analysis included 19 published neuroimaging studies on the neural substrates of discourse inferences (see Table 2) and 20 experiments reported in the 19 published articles on the neural substrates of logical problem-solving (see Table 3). The studies of the discourse comprehension group suggest various types of inferences, such as bridging inferences (e.g., Kuperberg et al., 2006; Virtue et al., 2008; Mason and Just, 2011; Kim et al., 2012), predictive inferences (e.g., Chow et al., 2008; Jin et al., 2009), and elaborative inferences (e.g., Yarkoni et al., 2008). Similarly, the 20 experiments of the logical reasoning group also covered various types of reasoning, such as inductive reasoning (e.g., Goel et al., 1997; Goel and Dolan, 2004), conditional reasoning (e.g., Canessa et al., 2005, 2014; Monti et al., 2007; Coetzee and Monti, 2018), and syllogistic reasoning (e.g., Goel et al., 1998; Jia et al., 2009; Rodriguez-Moreno and Hirsch, 2009). The studies also differed in many aspects, including the ways of stimulus presentation (i.e., visual or auditory) and response (i.e., reading or listening without an immediate response, or a response by pressing keys or buttons), neuroimaging methods (i.e., PET or fMRI), templates (i.e., MNI or Talairach), and software packages used for preprocessing and statistical analysis.

**Table 2:** Studies on the discourse inferences that were included in the meta-analysis.

**References**	**Scanning method**	**Template**	***N***	**Stimuli presentation**	**Response**	**Number of peaks**	**Contrast of interest**
Chow et al. (2008)	fMRI	Montreal Neurological Institute (MNI)	15	Visual	Button press (1 out of 2)	5	Predictive reading > Normal reading
Ferstl and von Cramon (2001)	fMRI	Talairach	12	Visual	Button press (1 out of 2)	2	Coherence > Incoherence
Ferstl and von Cramon (2002)	fMRI	Talairach	9	Auditory	Button press (1 out of 2)	12+5 ^a^	Logic coherence > Logic incoherence
Fletcher et al. (1995)	PET	Talairach	6	Visual	No response ^b^	5	1. Mental state stories > Unlinked sentences
						4	2. Physical stories > Unlinked sentences
Friese et al. (2008)	fMRI	MNI	13	Visual	Button press (1 out of 2)	1	Inference > Paraphrase
Jin et al. (2009)	fMRI	Talairach	15	Visual	No response	2	Predictive > Non-predictive Control
Kim et al. (2012)	PET	Talairach	10	Visual	Button press (1 out of 2)	6	1. Strong coherence > Control
						4	2. Weak coherence > Control
Kranjec et al. (2012)	fMRI	Talairach	18	Visual	Button press (1 out of 2)	14	Causality > Space and Time
Kuperberg et al. (2006)	fMRI	Talairach	15	Visual	Button press (1 out of 3)	8	Intermediately related > Highly related
						7	Intermediately related > Unrelated
Mason and Just (2011)	fMRI	MNI	10	Visual	Button press (1 out of 2)	17	1. Intentional inference > Control (sentence 2)
						1	2. Physical Inference > Control (sentence 2)
						5	3. Intentional inference > Control (sentence 3)
Monti et al. (2009)	fMRI	MNI	15	Visual	Button press (1 out of 2)	44	Inference > Grammar for linguistic arguments
Prado et al. (2015)	fMRI	MNI	20	Visual	Button press (1 out of 2)	8	Disjunctive stories > Control
Prat et al. (2011)	fMRI	MNI	18	Visual	Button press (1 out of 2)	18	Coherent > Incoherent
Robertson et al. (2000)	fMRI	Talairach	8	Visual	No response	2	More coherent > Less coherent
Siebörger et al. (2007)	fMRI	Talairach	14	Auditory	Button press (1 out of 4)	9	1. Distantly related > Unrelated
						1	2. Distantly related > Closely related
Virtue et al. (2006)	fMRI	Talairach	17	Auditory	No response	2	1. Implied > Explicit (verb point)
						2	2. Implied > Explicit (coherence break)
Virtue et al. (2008)	fMRI	Talairach	19	Auditory	No response	6	Predictable > Explicit
Xu et al. (2005)	fMRI	MNI	22	Visual	No response	17	Coherent narratives > Unconnected sentences
Yarkoni et al. (2008)	fMRI	Talairach	29	Visual	No response	10	Story > Scrambled

*N, number of participants*.

a *Ferstl and von Cramon (2002) additionally reported five foci in the extent of activations in medial prefrontal cortex (MPFC)*.

b *In the experiments marked “No response,” the participants did not respond right after stimulus presentation, but may provide delayed response after finishing a block or the scanning*.

**Table 3:** Studies on the logical inferences that were included in the meta-analysis.

**References**	**Scanning method**	**Template**	***N***	**Stimuli presentation**	**Response**	**Number of peaks**	**Contrast of interest**
Canessa et al. (2005)	fMRI	MNI	12	Visual	Button press (1 out of 4)	18	1. Descriptive reasoning > Baseline
						23	2. Social-exchange reasoning > Baseline
Canessa et al. (2014)	fMRI	MNI	14	Visual	Button press (1 out of 4)	26	1. Standard conditional rules > Baseline
						12	2. Switched conditional rules > Baseline
Coetzee and Monti (2018)	fMRI	MNI	20	Visual	Button press (1 out of 2)	30	Complex > Simple reasoning
Goel and Dolan (2001)	fMRI	MNI	14	Visual	Button press (1 out of 2)	12	Concrete reasoning > Concrete baseline
Goel and Dolan (2004)	fMRI	MNI	16	Visual	Button press (1 out of 2)	13	Reasoning > Baseline
Goel et al. (1997)	PET	Talairach	10	Visual	Button press (1 out of 2)	3	1. Deduction > Baseline
						6	2. Induction > Baseline
Goel et al. (1998)	PET	Talairach	12	Visual	Button press (1 out of 2)	4	1. Syllogism > Baseline
						5	2. Spatial relational > Baseline
						3	3. Nonspatial relational > Baseline
Goel et al. (2000)	fMRI	MNI	11	Visual	Button press (1 out of 2)	7	Content reasoning > Preparation
Goel et al. (2004)	fMRI	MNI	14	Visual	Button press (1 out of 2)	14	1. Reasoning > Baseline in unfamiliar environment
						5	2. Reasoning > Baseline in familiar environment
Goel et al. (2009)	fMRI	Talairach	17	Visual	Button press (1 out of 2)	10	Reason > Baseline
Jia et al. (2009)	fMRI	Talairach	11	Visual	Button press (1 out of 2)	8	Forward-chaining syllogism > Baseline
						11	Backward-chaining syllogism > Baseline
Knauff et al. (2002)	fMRI	Talairach	12	Auditory	Button press (1 out of 2)	16	Relational or conditional reasoning > Baseline
Liu et al. (2012)	fMRI	Talairach	14	Visual	Button press (1 out of 4)	16	1. Falsification > Irrelevance condition
						9	2. Non-falsification > Irrelevance condition
Monti et al. (2007) Exp1	fMRI	MNI	10	Visual	Button press (1 out of 2)	5	Complex > Simple deductions to block content
Monti et al. (2007) Exp2	fMRI	MNI	12	Visual	Button press (1 out of 2)	26	Complex > Simple reasoning
Monti et al. (2009)	fMRI	MNI	15	Visual	Button press (1 out of 2)	26	Inference > Grammar for logic arguments
Noveck et al. (2004)	fMRI	MNI	16	Visual	Button press (1 out of 3)	4	1. Modus Ponens> Baseline
						6	2. Modus tollens> Baseline
Osherson et al. (1995)	PET	Talairach	10	Visual	No response ^c^	8	1. Logical reasoning > Meaning
						8	2. Probabilistic reasoning > Meaning
Prado et al. (2010)	fMRI	MNI	13	Visual	Button press (1 out of 3)	5	1. Integrable > Non-integrable (Modus Tollens)
						7	2. Integrable > Non-integrable (relational syllogism)
Rodriguez-Moreno and Hirsch (2009)	fMRI	Talairach	11	Visual and Auditory	Button press (1 out of 2)	5	1. Reasoning > Control during the second premise
						9	2. Reasoning > Control during the conclusion

*N, number of participants*.

c *In the experiments marked “No response,” the participants did not respond right after stimulus presentation, but may provide delayed response after finishing a block or the scanning*.

### Meta-Analytic Approach

The GingerALE 3.0.2 software (http://www.brainmap.org) was used for conducting the meta-analysis of functional neuroimaging data (Eickhoff et al., 2009, 2012; Turkeltaub et al., 2012). The ALE approach is suitable for carrying out meta-analyses, for it treats the reported foci not as single points, but as centers for the three-dimensional (3D) Gaussian probability distributions that reflect the reliability of neuroimaging results (Eickhoff et al., 2009). For each experiment, we calculated a modeled activation (MA) map using the ALE method, which contained the Gaussian probability distributions of all reported foci of all contrasts-of-interest in this particular experiment. In the current meta-analysis, all included experiments were obtained from the different samples. The full-width half-maximum (FWHM) values of Gaussian distributions were determined by using the number of participants. Next, separate MA maps for each group of studies were combined into a single ALE map, and the ALE values were computed on a voxel-by-voxel basis (Eickhoff et al., 2012). The value of *p* of a particular ALE value was calculated by using the random-effects inference.

### Meta-Analysis Procedure

Reported foci in the contrasts-of-interest of each included experiment were extracted by two of the current authors independently (WW and WF). They re-examined and resolved rare disagreements. In total, there were 217 foci extracted from the studies on discourse understanding and 360 foci from the studies on logical problem-solving. All activation coordinates reported in the Talairach space were first converted into MNI space by using the GingerALE converter tool (Lancaster et al., 2007; Laird et al., 2010). Next, we separately performed an ALE analysis for each group of studies (discourse understanding vs. logical problem-solving) and obtained their respective ALE map. Based on the recommendations of Eickhoff et al. (2012, 2016), we evaluated the results of these single data set analyses using an uncorrected voxel-level threshold at *p* < 0.001 and a cluster-level threshold at *q* < 0.05 family-wise error (FWE) corrected. We applied 5,000 threshold permutations to derive cluster-level thresholds.

To determine the shared and separated neural correlates of discourse inferences and logical inferences, we conducted contrast analyses between the two groups of studies. Contrast analyses computed the voxel-wise difference between two thresholded ALE images calculated from the single data set analyses (Laird et al., 2005; Eickhoff et al., 2011). The conjunction was created by using the voxel-wise minimum value of these ALE images. We subtracted one input image from the other to generate two ALE contrast images. Considering the balance between sensitivity and specificity (Eickhoff et al., 2012), in the contrast data set analyses, we conducted a threshold of uncorrected *p* < 0.01 after 10,000 permutations and excluded the results with a volume size smaller than 200 mm^3^.

### Pattern Similarity Analyses

We used the Neurosynth Image Decoder (http://neurosynth.org/decode; Yarkoni et al., 2011) to further quantify the neural pattern similarity between the ALE maps and the patterns associated with the cognitive terms obtained from previous neuroimaging studies. More than 14,000 published studies have been included in the Neurosynth database by the time we conducted the decoding (December 2020). The reverse inference *z* map for each of the 1,307 terms has been automatically generated by using the Neurosynth database. The Pearson correlation coefficients (*r*) between the unthresholded ALE map and the reverse inference map for each term were calculated. The *r* values reflect the spatial correlation across voxels between the two maps. All maps were restricted by using a gray matter mask.

## Results

The current study analyzed 19 experiments on discourse inferences and 20 experiments on logical inferences to identify the neural correlates of these two cognitive processes, as well as the shared and distinct neural correlates of them. For the discourse comprehension group, participants were 285 healthy adults (~51% females) while for the logical problem group, participants were 264 healthy adults (41% females). All participants were right-handed with the exception of one study (Mason and Just, 2011), which did not report participants' handedness.

### Single Data Set Analyses

**Figure 2:**
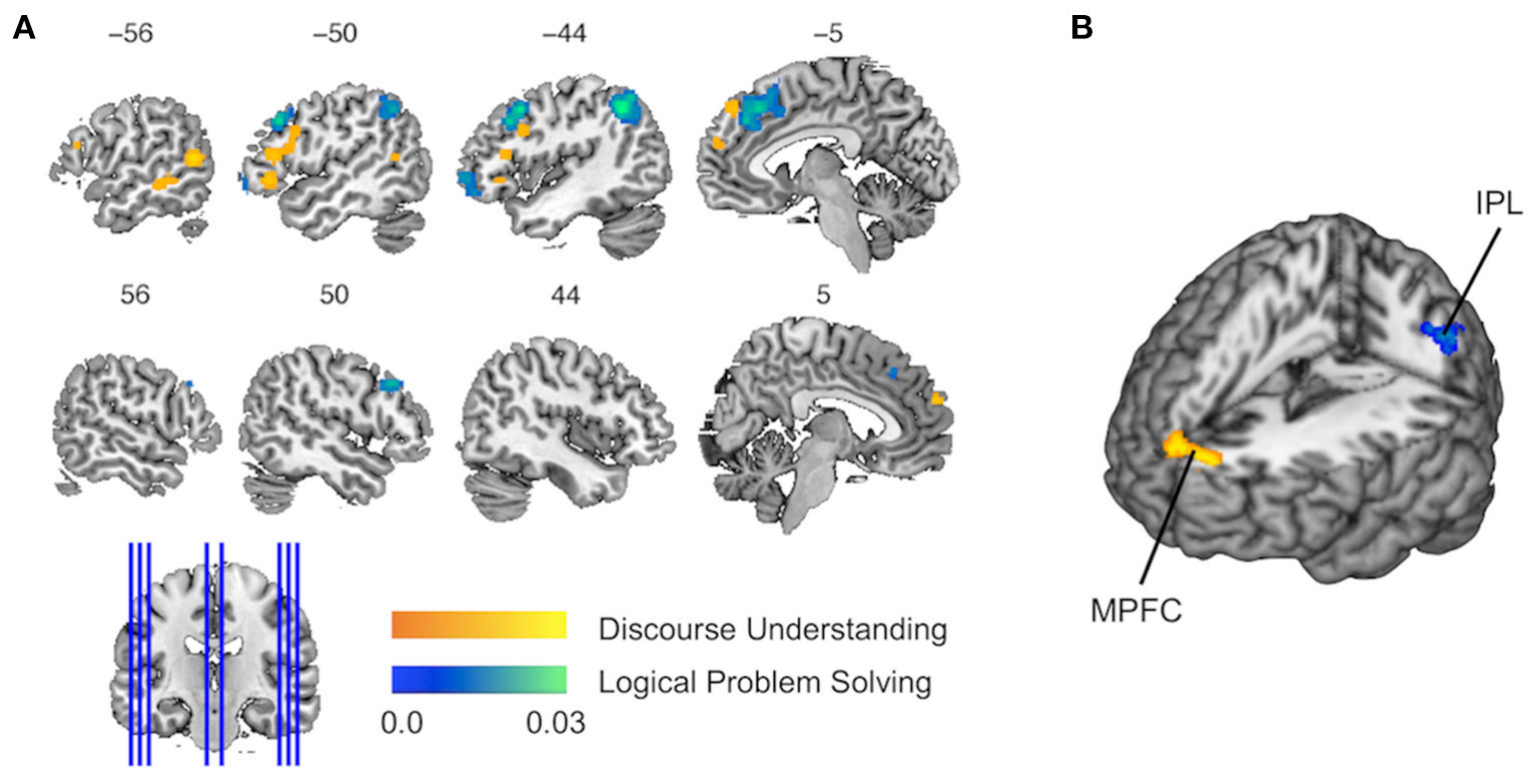
Results of single data set analyses and contrast data sets analysis. **(A)** The activation likelihood estimation (ALE) maps show the significant activations associated with discourse inferences (in orange) and logical inferences (in blue). **(B)** The contrast map shows the activation for the contrast of discourse inferences vs. logical inferences (in orange) and the activation for the reverse contrast (in blue). MPFC, medial prefrontal cortex; IPL, inferior parietal lobule.

The single data set analysis of discourse inferences revealed that six clusters of activations achieved significant convergence across the experiments (Figure 2A and Table 4). The pattern of activations showed left-hemisphere laterality. The largest cluster was observed in the pars triangularis and opercularis of the left IFG extending to the left precentral gyrus [Brodmann area (BA) 45/44/48]. Another cluster in the left IFG was found in the pars orbitalis extending to the pars triangularis (BA 47/45). Two other clusters were detected in the middle (BA 21) and posterior (BA 37/21) parts of the left MTG, respectively. Finally, two more clusters were in MPFC (BA 10 and 9).

**Table 4:** Regions are consistently activated across the experiments on discourse inferences and logical inferences.

**Location**	**BA**	**Peak coordinates**			**ALE value**	***z* value**	**Cluster (mm^**3**^)**
		**x**	**y**	**z**			
**DISCOURSE INFERENCES**							
L inferior frontal gyrus, pars tri/oper	45/44/48	−48	26	14	0.0196	4.82	2,264
L inferior frontal gyrus, pars orb/tri	47/44	−50	30	−4	0.0177	4.51	784
L middle temporal gyrus	37/21	−56	−56	10	0.0221	5.23	1,168
L middle temporal gyrus	21	−58	−34	−6	0.0152	4.08	776
L/R medial frontal cortex	10	6	60	24	0.0173	4.43	984
L medial frontal cortex	9	−8	48	46	0.0182	4.59	952
**LOGICAL INFERENCES**							
L medial frontal cortex	8/6/32	−6	30	46	0.0250	5.34	4,712
R medial frontal cortex	32/8	12	26	42	0.0241	5.20	976
L inferior parietal lobule	40/39	−44	−54	46	0.0316	6.28	4,512
L middle frontal gyrus	9/6/8	−42	14	44	0.0278	5.75	4,224
L middle/inferior frontal gyrus, pars orb	10/46	−40	54	−2	0.0244	5.26	2,048
R middle frontal gyrus	9/8	52	22	36	0.0228	5.01	960
**DISCOURSE INFERENCES** **∩** **LOGICAL INFERENCES**							
φ							
**DISCOURSE INFERENCES** **>** **LOGICAL INFERENCES**							
L/R medial frontal cortex	9	−4.9	56.6	19.7		3.43	968
**LOGICAL INFERENCES** **>** **DISCOURSE INFERENCES**							
L inferior parietal lobule	40	−50	−46	50		3.06	576

*All reported coordinates are in the MNI space. L, left; R, right; BA, Brodmann area*.

The single data set analysis of logical inferences revealed six clusters of activations, which achieved significant convergence across the experiments (Figure 2A and Table 4). The largest cluster and another cluster located in MPFC extending to the anterior cingulate cortex (BA 8/32/6). The second largest cluster was found in the left IPL (BA 40/39). Two other clusters were observed in MFG extending to the precentral gyrus (BA 9, 8, and 6) bilaterally. One more cluster covered the pars orbitalis of the left MFG extending to IFG (BA 46/10).

### Contrast Data Sets Analysis

The contrast data sets analysis did not reveal any significant activations that was commonly recruited by discourse inferences and logical inferences. When conducting a liberal threshold (*p* < 0.05 and volume > 50 mm^3^), we still observed no activation.

Compared to logical inferences, discourse inferences elicited a significant activation in MPFC (BA 9; see Figure 2B in orange, Table 4) whereas, compared to discourse inferences, logical inferences more strongly activated a brain region in the left IPL (BA 40; see Figure 2B in blue, Table 4).

### Pattern Similarity Analyses

**Figure 3:**
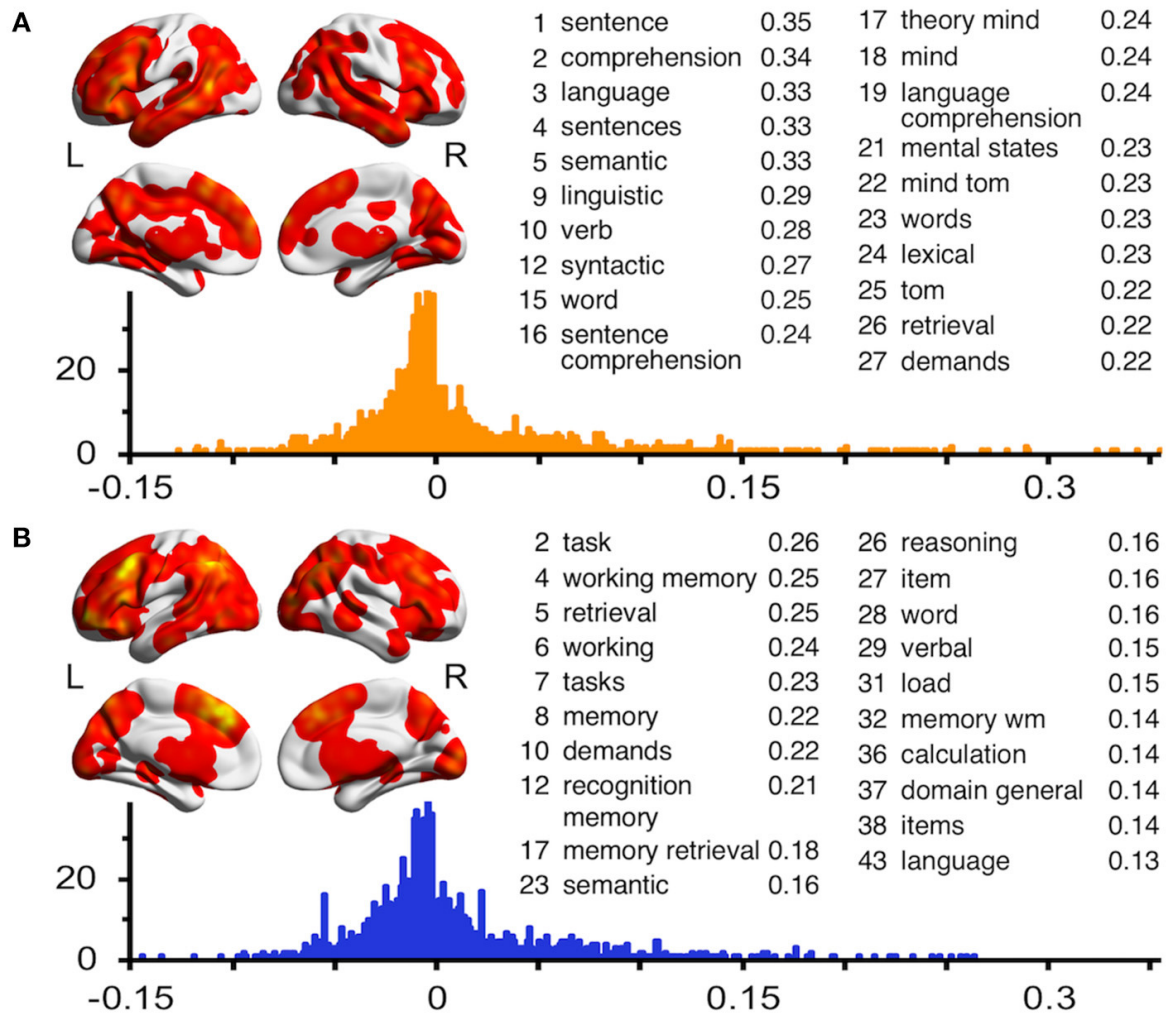
Results of neural pattern similarity analyses using the ALE map of discourse inferences **(A)** and logical inferences **(B)**. The unthresholded ALE maps are visualized on a surface rendering of the smoothed ICBM152 template. The histograms represent the frequency distributions of the Pearson's *r* between the ALE map and the *z* map for each term in the Neurosynth database. For each ALE map, the embedded table lists the most highly correlated cognitive or psychological terms ranked by *r* values. Terms associated with brain regions are not included in these tables.

To further investigate the cognitive processes that supported discourse inferences and logical inferences, we calculated the spatial correlations between the ALE map and the neural pattern for each term generated by using the Neurosynth database (see Figure 3). The mean *r* value was 0.009 (± SD = 0.061) for discourse inferences and 0.005 (±0.050) for logical inferences. The results showed that the discourse inferences were primarily related to the terms about language processing (e.g., sentence, comprehension, and language) and theory-of-mind processing (e.g., theory mind, mind, and mental states). Meanwhile, this process was also related to the terms associated with memory such as retrieval (*r* = 0.22), memory (0.143), and working memory (0.142), and executive function such as demand (0.217), task (0.140), and tasks (0.134). In contrast, logical inferences were mainly related to the terms about executive function (e.g., task, tasks, and demands) and memory (e.g., working memory, retrieval, and memory). In addition, this process was also related to the terms associated with language comprehension, like semantics (0.161), word (0.157), and language (0.129). These findings indicated that both discourse inferences and logical inferences need the support of the cognitive processes associated with memory, executive function, and language, but their extent of participation in the two types of inferential processing was different. Furthermore, discourse inferences are additionally associated with theory-of-mind processing.

## Discussion

The aims of the present meta-analysis study were to identify the neural substrates of discourse inferences and to compare it with those neural substrates of logical inferences. Using the ALE approach (Eickhoff et al., 2009, 2012), we analyzed 19 neuroimaging experiments on discourse inferences and 20 experiments on logical inferences and separately identified the commonly activated brain regions across the experiments for each process. Our study demonstrates that discourse inferences mainly engage a left-lateralized frontotemporal brain system whereas logical inferences rely on a non-overlapping brain system in the frontal and parietal cortex. The pattern similarity analyses also revealed that the subprocesses involved in the two inferential processing are different.

### Causal Inferences in Discourse Understanding

Previous studies on discourse comprehension have shown various results of neural correlates underlying causal inferences. This meta-analytic study reveals that the left IFG, left middle and posterior MTG, and bilateral MPFC are congruently activated by using discourse inferences across the experiments.

In the stage of study selection, we adopted a relatively strict criterion and excluded the studies using low-level baseline conditions such as fixation presentation. After subtracting adequate baseline conditions, discourse inferences still activated the core brain regions of language processing (i.e., IFG and MTG), which is congruent with a previous meta-analysis based on eight studies (Yang et al., 2019). Specifically, the left IFG was well-known as a core region of syntactic and semantic processing such as grammatical categorization (Ni et al., 2000), syntax parsing (Friederici and Kotz, 2003), semantic information retrieval (Wagner et al., 2001), semantic selection (Thompson-Schill et al., 1997; Kan and Thompson-Schill, 2004), and semantic unification (Hagoort et al., 2004; Zhu et al., 2012) while the left MTG was found to be associated with semantic processing (Xu et al., 2005; Binder et al., 2009; Visser and Lambon Ralph, 2011), especially a fine semantic activation that supports the processing of semantic integration [see Jung-Beeman (2005) for a review]. Based on the construction-integration model, discourse comprehension involves the process of constructing a discourse representation, which mainly relies on a conceptual or propositional net (Kintsch, 1988). When individuals need to draw discourse inferences, associative information will be elaborated and added to the existing discourse representation. Meanwhile, plenty of studies have demonstrated that individuals' long-term memory and working memory play important roles in discourse inferences (Keenan et al., 1984; Myers et al., 1987; Myers and Duffy, 1990). Thus, in the current study, the congruently enhanced activations in the left IFG and MTG during discourse inferences may reflect the processing of retrieving and selecting additional information from a semantic memory, and the processing of integrating them together to construct higher-order discourse representations in the working memory. Being consistent with this idea, the pattern similarity analysis revealed that discourse inferences would recruit the processing of language and memory.

Similarly, two clusters of activations within the anterior and dorsal MPFC (BA 10/9) also contribute to discourse inferences. MPFC has been reported in association with various high-order cognitive processes such as theory-of-mind processing (Van Overwalle and Baetens, 2009; Koster-Hale and Saxe, 2013), coherence evaluation (Chow et al., 2008), and inductive reasoning (Ferstl and von Cramon, 2002; Siebörger et al., 2007). Previous studies on discourse comprehension have suggested that the engagement of MPFC reflects controlled, strategic, and higher-order inferential processes (Ferstl and von Cramon, 2001, 2002; Kuperberg et al., 2006). We consider this idea as the most reasonable explanation of the role of MPFC in discourse inferences. However, as shown in the present pattern similarity analysis, discourse inferences may engage the theory-of-mind-related inferential processing. This may be caused by the fact that we did not distinguish the contents of causal inferences; that is, this meta-analysis included the studies involving both physical and intentional inferences (e.g., Fletcher et al., 1995; Mason and Just, 2011).

### Causal Inferences in Logical Problem-Solving

This meta-analysis found that a frontoparietal network, including the left IFG, bilateral MFG, MPFC (extending to the anterior cingulate cortex), and left IPL, would be consistently recruited by using logical inferences across the studies. In this study, we included neuroimaging experiments on verbal and content-based causal inferences across inductive and deductive reasoning, conditional and syllogistic inferences. Although the current meta-analysis is different from the previous meta-analyses in study selection, the results are essentially consistent with the previous findings of logical inferences (Prado et al., 2011; Wertheim and Ragni, 2020). Thus, our results further confirmed the association between verbal logical reasoning and activity in the left-lateralized frontoparietal network.

The lateral prefrontal cortex, including IFG and MFG, is related to working memory load (Rypma and D'Esposito, 1999; Fletcher and Henson, 2001; Jia et al., 2009). Furthermore, previous studies have demonstrated that content-based inferences additionally require the retrieval and application of world knowledge (Goel et al., 2000; Wertheim and Ragni, 2020). Thus, in logical problem-solving, the engagement of IFG and MFG reflects the processing of retrieving, maintaining, and refreshing information in memory. Meanwhile, IPL has been known to be linked with domain-general executive functions (Ye and Zhou, 2009a,b; Duncan, 2010). Such explanations are confirmed by using the results of the pattern similarity analysis, which suggested that the activation pattern of logical inferences is spatially correlated to that of memory and executive control.

### Dissociation of Causal Inferences in Discourse Understanding and Logical Problem-Solving

In this study, the single-study analyses showed that discourse inferences and logical inferences elicit basically different brain activation patterns. Moreover, the conjunction analysis between the two data sets did not yield any significant overlapping in terms of brain activation, further strengthening this observation. More specifically, although both discourse inferences and logical inferences recruit activations in the left lateral and MPFC (IFG and MPFC), these activations did not overlap.

First, as shown in our results, discourse inferences activated more posterior and ventral parts of the left lateral prefrontal cortex while logical inferences activated more anterior and dorsal parts (see Figure 2A). A similar pattern of results has been found by using a previous meta-analysis on analogical reasoning (Hobeika et al., 2016). In their work, the contrast of semantic vs. visuospatial analogy revealed an activation located in the posterior and ventral part of the left IFG while the contrast of visuospatial vs. semantic analogy revealed clusters in the anterior region of the left IFG and MFG, dorsal to the semantic analogy region. Moreover, Monti et al. (2009) also reported similar results that linguistic inferences recruited an activity in the vicinity of Broca's area (left BA 44/45) while logical inferences recruited an activity in the rostrolateral section of the left middle and IFG (BA 10/47). According to the assumptions of hierarchical models, the organization of the prefrontal cortex along the rostro-caudal axis is based on the content of a working memory representation (Badre, 2008; Badre and D'Esposito, 2009; Christoff et al., 2009). More specifically, more anterior and dorsal parts of the lateral prefrontal cortex support progressively more abstract representations in working memory while more posterior and ventral parts support more concrete representations. Thus, we believed that the activity in the left lateral frontal cortex is responsible for constructing concrete representations in working memory during discourse inferences, but for constructing abstract representations during logical inferences. The essentiality of representations has been stressed by using both classic models of discourse inferences [e.g., the construction-integration model (Kintsch, 1988), the constructionist theory (Singer, 1994)], and models of logical inferences [e.g., the mental model theory of reasoning (Johnson-Laird, 1999)].

Second, our study showed that both discourse inferences and logical inferences activated the clusters in the dorsal MPFC, but the activation cluster of discourse understanding is anterior to a larger activation cluster of logical problem-solving. Previous findings of the neural bases of conflict control demonstrated that distinct brain regions in dorsal MPFC respond to conflicts arising at different information inputs (Jost et al., 2012). Hence, the activity in the dorsal MPFC may reflect the selection and coordination of multiple task demands in discourse inferences and logical inferences. In discourse comprehension, this region may be central to the establishment of coherence under uncertainty (Ferstl and von Cramon, 2001; 2002). Meanwhile, in logical problem-solving, this region may enable individuals to convert premises into conclusions when multiple rules are available (Monti et al., 2009; Rodriguez-Moreno and Hirsch, 2009). In addition, the bilateral anterior portions of MPFC are exclusively recruited by using discourse inferences. This region may be involved in the connection between conceptual information and their affective or social meaning (see Roy et al., 2012 for a review). These results suggested that the affective and social information might be more involved in discourse inferences than in logical inferences.

Overall, in the current study, we found that the cognitive processes of memory and executive control were involved in both discourse inferences and logical inferences whereas the neural bases underlying the two types of causal inferences were completely separate: the discourse inferences relied more on language processing and theory-of-mind-related inferential processing. In combination with these findings, we speculated that discourse inferences and logical inferences may recruit different subcomponents of the cognitive processes associated with memory and executive control. The dissociation of the two types of causal inferences originates from different inference contents. In discourse inferences, the construction of a discourse representation in the working memory mainly depends on one's semantic knowledge and social interaction experience. Relatively, although logical inferences are also based on verbal stimuli in the current meta-analysis, such inferences rely more on abstract representations in the working memory.

Notably, by directly comparing linguistic inferences and logical inferences in the same population, Monti et al. (2009) revealed that the activations of linguistic inferences and logical inferences overlapped in inferior/middle frontal gyri and superior/IPLs, which has been reported to be associated with the working memory and executive functions (Rypma and D'Esposito, 1999). Although we found that the cognitive processes of memory and executive control were involved in both discourse inferences and logical inferences, our meta-analysis did not identify any co-activation between both types of inferences. The inconformity between our results and the results of Monti et al. (2009) could be caused by several reasons. First, this meta-analysis incorporates a number of experiments, which exhibit heterogeneity in various aspects. With the exception of Monti et al. (2009), the studies included in the current meta-analysis do not investigate both discourse inferences and logical inferences, and each group of studies included several types of inferences as we mentioned in the Section “Method.” Given that, it is hard for the current meta-analysis to detect the conjunction that only appears when a certain set of conditions are satisfied. Likewise, since meta-analyses are limited to the studies available, we could not rule out the possibility that the lack of significant co-activations is due to the differences in the aspects of the subject, task, etc., rather than differences between discourse inferences and logical inferences. In addition, with the limited number of available studies and the hard-to-control variation within each group of studies, it should also be considered that this absence of co-activations may be caused by the fact that the statistical power is insufficient. Thus, more data points need to be accumulated in order to obtain objective and accurate results in the future. Future research is expected to investigate what factors would influence the activity pattern in the inferior/middle frontal cortex and superior/IPLs during discourse inferences and logical inferences.

## Conclusion

Our meta-analysis identified the neural correlates of both discourse inferences and logical inferences. Discourse inferences recruit a left-lateralized frontotemporal brain system, including the left IFG, left middle and posterior MTG, and bilateral MPFC while logical inferences engage a frontoparietal brain system, consisting of the left IFG, bilateral MFG, dorsal MPFC, and left IPL. Furthermore, this meta-analysis contributes to the question of whether the two types of inferential processing have shared neural bases by revealing that they rely on the activities in separate brain regions. Considering that the cognitive processes of memory and executive control are involved in both inferential processes, the dissociation suggests that discourse inferences and logical inferences use the information from different sources to construct mental representations in the working memory. The current meta-analysis identifies the neural correlates that are consistently activated by using discourse inferences and logical inferences across the studies. These findings provide reliable quantitative evidence and extend current knowledge for the neural correlates underlying the two types of causal inferences. The dissociation of discourse inferences and logical inferences may remind that the analogy between the two should be carefully considered. In addition, this meta-analysis confirms the important roles of the working memory and executive function in both discourse inferences and logical inferences, which may be of assistance to build and extend cognitive models to account for causal inferences.

## Data Availability Statement

The raw data that support the conclusions of this study are available on request from the corresponding authors, without undue reservation.

## Author Contributions

WF: conceptualization, methodology, formal analysis, writing—original draft, and visualization. WW: formal analysis, investigation, and writing—original draft. JL and ZW: investigation and writing—original draft. LT: writing—original draft and visualization. LF: writing—review and editing. All authors contributed to the article and approved the submitted version.

## Conflict of Interest

The authors declare that the research was conducted in the absence of any commercial or financial relationships that could be construed as a potential conflict of interest.
